# Assessment of Titanate Nanolayers in Terms of Their Physicochemical and Biological Properties

**DOI:** 10.3390/ma14040806

**Published:** 2021-02-08

**Authors:** Michalina Ehlert, Aleksandra Radtke, Katarzyna Roszek, Tomasz Jędrzejewski, Piotr Piszczek

**Affiliations:** 1 Faculty of Chemistry, Nicolaus Copernicus University in Toruń, Gagarina 7, 87-100 Toruń, Poland; m.ehlert@doktorant.umk.pl; 2 Nano-implant Ltd., Gagarina 5/102, 87-100 Toruń, Poland; 3 Faculty of Biological and Veterinary Sciences, Nicolaus Copernicus University in Toruń, Lwowska 1, 87-100 Toruń, Poland; kroszek@umk.pl (K.R.); tomaszj@umk.pl (T.J.)

**Keywords:** titanate nanolayers, structure, surface morphology, bone-like apatite, biointegration activity

## Abstract

The surface modification of titanium substrates and its alloys in order to improve their osseointegration properties is one of widely studied issues related to the design and production of modern orthopedic and dental implants. In this paper, we discuss the results concerning Ti6Al4V substrate surface modification by (a) alkaline treatment with a 7 M NaOH solution, and (b) production of a porous coating (anodic oxidation with the use of potential U = 5 V) and then treating its surface in the abovementioned alkaline solution. We compared the apatite-forming ability of unmodified and surface-modified titanium alloy in simulated body fluid (SBF) for 1–4 weeks. Analysis of the X-ray diffraction patterns of synthesized coatings allowed their structure characterization before and after immersing in SBF. The obtained nanolayers were studied using Raman spectroscopy, diffuse reflectance infrared Fourier transform spectroscopy (DRIFT), and scanning electron microscopy (SEM) images. Elemental analysis was carried out using X-ray energy dispersion spectroscopy (SEM EDX). Wettability and biointegration activity (on the basis of the degree of integration of MG-63 osteoblast-like cells, L929 fibroblasts, and adipose-derived mesenchymal stem cells cultured in vitro on the sample surface) were also evaluated. The obtained results proved that the surfaces of Ti6Al4V and Ti6Al4V covered by TiO_2_ nanoporous coatings, which were modified by titanate layers, promote apatite formation in the environment of body fluids and possess optimal biointegration properties for fibroblasts and osteoblasts.

## 1. Introduction

The wide use of metal implants in the treatment of bone injuries and other diseases requiring their surgical implantation, which can support tissue regeneration and facilitate bone differentiation and development, is one of the most important directions in the development of modern medicine. From the technological point of view, the selection of materials used in the construction of implants depends on the functions that they will perform in the human body [1,2,3,4,5,6]. In the case of metallic implants, especially important is their surface, which should exhibit biological compatibility in the interaction with tissues and body fluids in the human body [7,8,9,10,11,12,13] Good mechanical properties and appropriate bioactivity of titanium and its alloys contributed to the wide use of these materials in the production of modern bone implants and bone and tissue scaffolding [10,11,12,14]. In this case, the processes aimed at creating a permanent connection of the implant surface with the bone (i.e., osseointegration) are also of key importance [1,15]. The results of previous investigations revealed that the osseointegration process depends on the surface topography, the roughness, the structure, and the chemical composition [3,13,14,16,17]. The surface chemical composition of the material, which directly influences parameters such as surface energy, corrosivity, and wettability, is especially important [1,2,3,18,19,20,21]. Therefore, numerous surface modification techniques are currently used to achieve the desired physical, chemical, or biological properties of the material [22,23,24]. The surface modification of substrates made from titanium and its alloys by alkaline treatment is an increasingly commonly used technique [25,26,27]. It involves the dissolution in alkaline solution of the TiO_2_ passive layer and the production of negatively charged hydrates (HTiO_3_^−^ ⋯ nH_2_O) on the substrate surfaces. Sodium ions in the aqueous solution are combined with the negatively charged species, forming a sodium titanate hydrogel layer. The further heat treatment leads to dehydration and compaction of the hydrogel layer, which leads to the formation of the stable amorphous or crystalline titanate layer [28,29,30]. Various kinds of sodium titanates have been synthesized, which consist of negatively charged sheets made from TiO_6_^8−^ octahedra, sharing corners and edges with each other [31,32,33]. Depending on the sodium ion content, the titanates form different structures (cage, tunnel, or layered), resulting in differences in their properties. The alkali-sodium titanates (Na_2_Ti_n_O_2n+1_), where *n* = 3 or 8, crystallize in a monoclinic system. Such materials for *n* = 3 or 4 consist of (Ti_3_O_7_)^2−^ layers held together with exchangeable alkali sodium ions (Na^+^). At lower sodium contents, Na_2_Ti_6_O_13_, Na_2_Ti_7_O_15_, or Na_2_Ti_8_O_17_ with a tunnel structure is formed. This structure exhibits a good chemical stability and a high insulating ability [32,33,34,35]. The titanate hydrogels attract much attention due to their improved biological and mechanical properties, as well as their high corrosion resistance. As such, sodium titanate composites are widely applied in orthopedics, as well as in gas sensors as a reference electrode; they are also used as photocatalysts and electrochemical capacitors [24,34,36,37,38,39,40]. In addition, to intensify the biological activity of the material, it is desirable to produce a coating that is capable of forming apatite on its surface in the body fluid environment. It is important that the resulting layer does not delaminate [15,36].

It should be noted that the sodium titanate hydrogel layer formed on the surface of modified Ti6Al4V substrate could stimulate the formation of apatite coatings in appropriate conditions [41,42,43,44]. The sodium titanates in an environment of simulated body fluid (SBF) solution (as well as in body fluids) form Ti–OH groups on their surfaces as a result of Na^+^ ion exchange with H_3_O^+^ ions present into the solution. The pH (in the surrounding fluid) increases and the negatively charged Ti–OH groups combine selectively with positively charged Ca^2+^ to form an intermediate apatite nucleation product, which is calcium titanate. Then, the Ca^2+^ cations accumulate on the surface and combine with phosphate ions, and the amorphous calcium phosphate formed in this way spontaneously transforms into apatite [28,38,43,44,45,46,47]. The above-described mechanism is important from the medical point of view. The bone partially consists of non-stoichiometric, inorganic calcium phosphate minerals; therefore, one of the ways to evaluate the bioactivity of a material is to study the spontaneous formation of apatite on its surface, while it is immersed in a physiological medium. The concentration of ions in simulated body fluid (SBF) is almost equal to the concentration of ions in human blood plasma; therefore, it can adequately reproduce the in vivo apatite formation [30,48,49,50,51]. On the other hand, these processes lead to the formation of an apatite layer linked with the substrate surface via a chemical bond, thereby rendering it more resistant to abrasion and delamination.

Considering the results of these investigations, we decided to carry out research on the applicability of the alkali-sodium treatment method of titania nanoporous coatings (TNT), which were produced during the anodization of Ti6Al4V, and to compare obtained results with those obtained for alkali-sodium-treated Ti6Al4V. Analysis of previous reports showed that, in the case of alkali-sodium-treated titania nanoporous coatings, their physicochemical and biological properties have not yet been analyzed and, hence, they can constitute an important new element of knowledge about surface modifications of titanium alloys.

Research on the stimulation and acceleration of bone tissue regeneration, stimulation of osteopromotional cells, and improvement of interlayer adhesion is of particular importance to us. The development of a sodium titanate layer-producing method on the surface of a (Ti6Al4V/nanoporous TiO_2_) system, determination of its physicochemical properties, and estimation of its biological activity are especially significant issues. The results discussed in this paper provide a basis for designing biocompatible materials, which can be used in the construction of titanium alloy implants with high osseointegration activity.

## 2. Materials and Methods

### 2.1. Sample Preparation

Commercially available titanium alloy (Ti6Al4V foil (marked as T), grade 5, 99.7% purity, 0.20 mm thick (Strem Chemicals, Inc., (Bischheim, France), 6 mm × 60 mm pieces and 10 mm × 60 mm pieces) was used as the substrate. Prior to alkali treatments, the specimens were polished and sonicated consecutively in acetone, ethyl alcohol, and deionized (DI) water, each for 10 min. Then they were air-dried at room temperature. In addition, some of the samples were anodized. The T was used as the anode and platinum foil was used as the cathode. Electrodes were placed 2 cm apart. The electrolyte consisted in an aqueous electrolyte solution—0.3% HF. The anodization potential was kept constant at 5 V for 20 min. Samples of the Ti6Al4V/nanoporous TiO_2_ system (marked as T5), which were produced during the anodizing, were rinsed with deionized water and dried in an argon stream.

### 2.2. Alkali-Sodium Surface Treatment of Samples

The prepared T and T5 samples were immersed in a 7 M NaOH solution at 65 °C for 48 h. After alkali-sodium treatment, the obtained samples were washed with distilled water, and dried at 40 °C for 24 h in an incubator. The specimens were marked as T-S and T5-S, respectively. All samples prepared for chemical and biological analyses were autoclaved using an IS YESON YS-18L (Yeson, Ningbo, China) at 123 °C, *p* = 120 kPa, *t* = 20 min.

### 2.3. Apatite-Forming Ability

After the alkali-sodium treatment, the samples with the dimension of 10 mm × 10 mm × 0.20 mm (T-S, T5-S, and T as a control) were immersed in SBF at a constant temperature of 36.5 °C for 7, 14, 21, and 28 days, and each was kept in a vertical position inside polypropylene tubes. We used SBF as a standard solution to detect the apatite formation on the surfaces of the prepared samples. The SBF solution has an ionic concentration nearly equal to human blood plasma. One liter of SBF was prepared according to ISO/FDIS 23317:2007(E) and Kokubo’s formulation by dissolving reagents given in the required order of dissolution (8.035 g NaCl, 0.355 g NaHCO_3_, 0.225 g KCl, 0.231 g K_2_HPO_4_·3H_2_O, 0.311 g MgCl_2_·6H_2_O, 0.292 g CaCl_2_, and 0.072 g Na_2_SO_4_ in DI water and buffering at pH 7.40 with Tris (tris(hydroxymethyl)aminomethane) and 1.0 M HCl at 36.5 °C). After immersing in the SBF, specimens were taken out from the solution, cleaned in deionized water, and dried at room temperature before characterization [52,53]. The specimens were marked as T-S/Ca and T5-S/Ca (after 7 days of immersion) and T-S/HA and T5-S/HA (after 14, 21, and 28 days of immersion).

### 2.4. Surface Characterization

Morphological evaluation of the T and T5 surfaces before and after the alkali-sodium treatment and the immersion in SBF was performed with a Quanta scanning electron microscope with field emission (SEM, Quanta 3D FEG, Huston, TX, USA). The chemical composition of the titanate layer after the alkali-sodium treatment and immersion in SBF was carried out using an energy-dispersive X-ray spectrometer (EDS, Quantax 200 XFlash 4010, Bruker AXS, Karlsruhe, Germany). The structure of the produced layers was estimated using Raman spectroscopy (RamanMicro 200 PerkinElmer (PerkinElmer Inc., Waltham, MA, USA) (*λ* = 785 nm)) and diffuse reflectance infrared Fourier transform spectroscopy (DRIFT, Spectrum 2000, PerkinElmer Inc., Waltham, MA, USA). The crystalline structure analysis of all produced layers was done using X-ray diffraction (XRD; PANalytical X’Pert Pro, PANalytical B.V., Almelo, The Netherlands MPD X-ray diffractometer using Cu-K alfa radiation, grazing incidence angle mode–GIXRD; the incidence angle was equal to 1°).

### 2.5. Contact Angle

The contact angle of water and diiodomethane on the samples was measured at room temperature using a goniometer (DSA 10 Krüss GmbH, Hamburg, Germany) with drop shape analysis software (ADVANCE, Krüss software, Krüss GmbH, Hamburg, Germany) with a drop volume of 3 µL for deionized water and 4 µL for diiodomethane onto each sample. All reported contact angles on the surface of the sample are the average of three samples from each series.

### 2.6. Cell Lines

L929 mouse fibroblasts were obtained from the American Type Culture Collection (Manassas, VA, USA). The cells were grown in complete Roswell Park Memorial Institute (RPMI) 1640 medium (with l-glutamine) supplemented with 10% fetal bovine serum (FBS), 100 IU/mL penicillin, and 100 µg/mL streptomycin.

MG-63 human osteoblast-like cells were purchased from the European Collection of Authenticated Cell Cultures (Salisbury, UK). The MG-63 cells were cultured in Eagle’s Minimum Essential medium (EMEM) supplemented with 10% FBS, 2 mM l-glutamine, 1 mM sodium pyruvate, nonessential amino acids, and antibiotics (100 U/mL penicillin, 100 μg/mL streptomycin).

Adipose-derived human mesenchymal stem cells (ADSCs) were purchased from PromoCell and cultured in Mesenchymal Stem Cell Growth Medium^®^ supplemented with 10% Supplement Mix^®^ (PromoCell GmbH, Germany), 100 U/mL penicillin, and 100 µg/mL streptomycin.

All cell lines were cultured at 37 °C in a humidified atmosphere with 5% CO_2_ and passaged at 70–80% confluency using a 0.04% trypsin ethylenediaminetetraacetic acid (EDTA) solution (ADSC cells), 0.25% trypsin-EDTA solution (MG-63 cells), or cell scraper (L929 fibroblasts). All compounds used for cell culture were purchased from Sigma-Aldrich (Darmstadt, Germany).

### 2.7. Cell Viability

The effect of the specimens on the cell viability was determined with the 3-(4,5-dimethylthiazol-2-yl)-2,5-diphenyltetrazolium bromide (MTT) assay. The cells (density 1 × 10^4^ cells/well) were cultured on autoclaved scaffolds placed in 24-well plates for 24, 72, and 120 h. After incubation, the specimens were washed with phosphate-buffered saline (PBS). Then, MTT solution (0.5 mg/cm^3^) was added and the samples were incubated for 3 h at 37 °C, and the resulting purple formazan crystals were dissolved in dimethyl sulfoxide (DMSO). The optical density at 570 nm (with a reference wavelength of 630 nm) was measured using a Synergy HT Multi-Mode microplate reader (BioTek; Winooski, VT, USA). The scaffolds incubated without the cells were used as blank samples. The results derived from five independent experiments.

### 2.8. Cellular Morphology

Scanning electron microscopy (SEM; Quanta 3D FEG; Carl Zeiss, Göttingen, Germany) was used to assess the changes in cell morphology. After the respective incubation time of cells on the scaffolds, the samples were rinsed with PBS and fixed in 2.5% *v*/*v* glutaraldehyde. After that, the specimens were dehydrated in graded series of ethanol concentrations for 10 min. Lastly, the samples were dried in vacuum-assisted desiccators overnight before the SEM analysis.

### 2.9. Statistical Analysis

All values from the MTT assay are reported as the means ± standard errors of the means (SEMs) and were determined by analysis of variance followed by Bonferroni multiple comparisons test, with the level of significance set at *p* < 0.05. Statistical analyses were done using GraphPad Prism 7.0 (La Jolla, CA, USA)

## 3. Results

### 3.1. Surface Characterization

**Figure 1:**
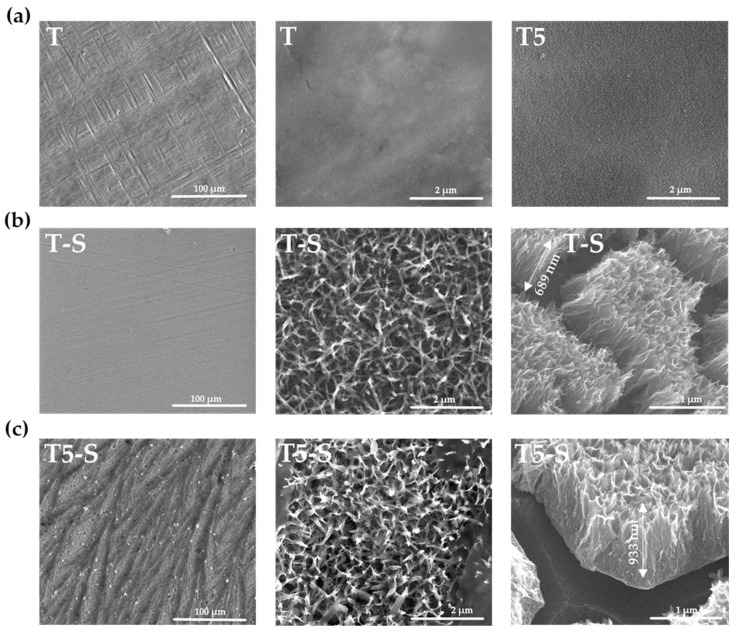
SEM images of the surface morphology and cross-sections of the T and T5 (**a**), T-S (**b**), and T5-S (**c**) samples.

The alkali-sodium treatment of the Ti6Al4V alloy (T) substrate in 7 M NaOH solution by 48 h led to the formation of the uniform coatings (T-S). The morphology changes of studied alloy samples before and after their immersion in alkali-sodium solution are presented in Figure 1a,b. The SEM cross-section images (Figure 1b) revealed that the produced coating thickness was 685 ± 5 nm. The immersion of the T5 sample (Ti6Al4V/TiO_2_ nanoporous system, which was produced by anodic oxidation of the Ti6Al4V substrate according to an earlier described procedure [3,22]) in the same conditions led to the formation of a three-dimensional network of tangled nanorods and nanofibers (Figure 1c). Analysis of SEM cross-section images proved that the thickness of T5-S system was 933 ± 3 nm.

**Figure 2:**
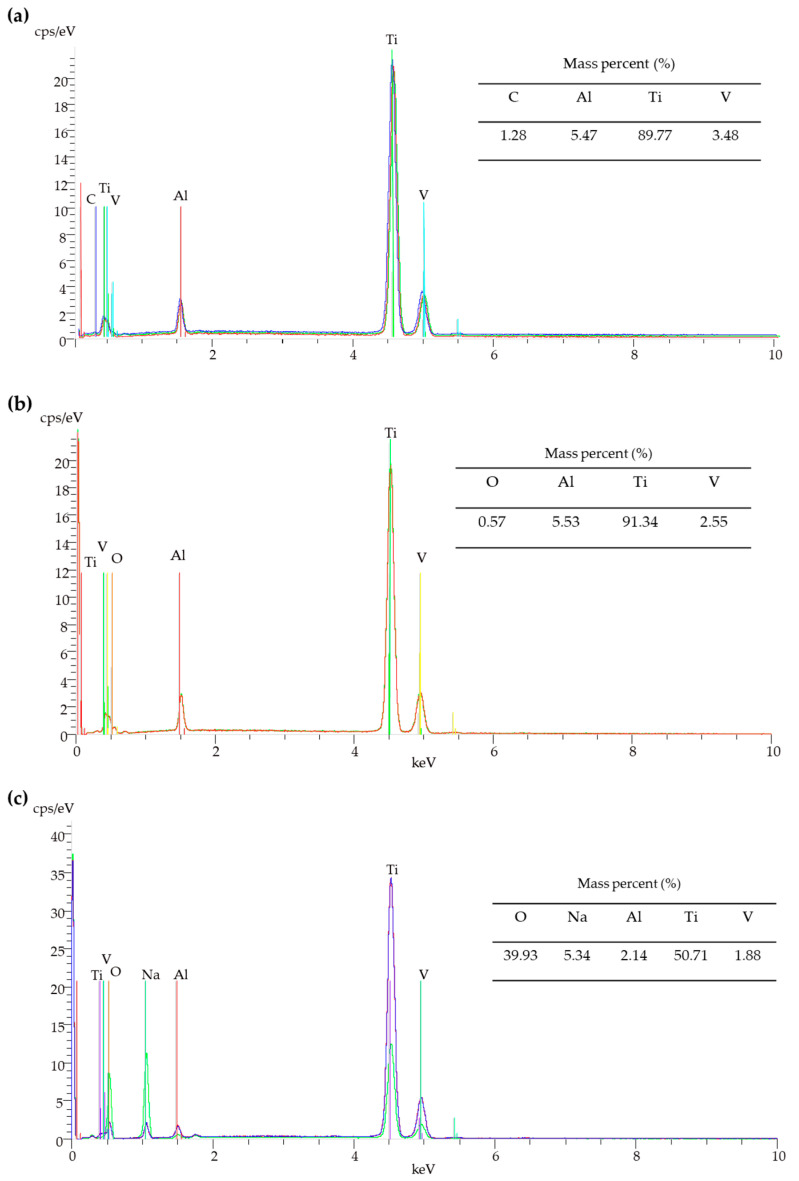

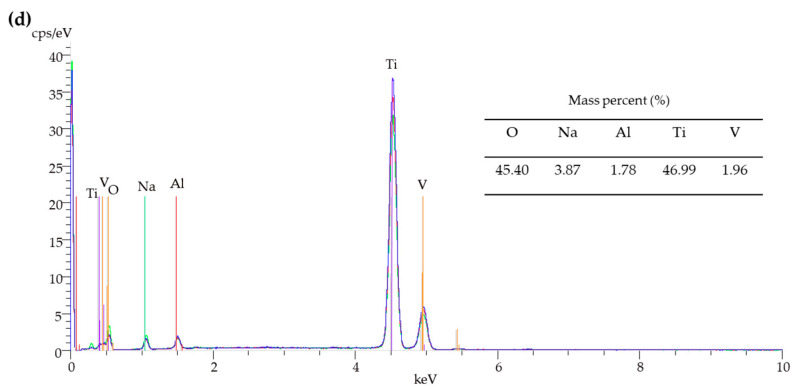
Energy-dispersive spectroscopy (EDS) spectra and quantitative data of the T (**a**), T5 (**b**), T-S (**c**), and T5-S (**d**) samples.

The use of scanning electron microscopy with energy-dispersive spectroscopy (SEM/EDS) method allowed confirming the presence of Na^+^ on the surface of the samples after alkaline treatments (T-S and T5-S) (Figure 2c,d).

**Figure 3:**
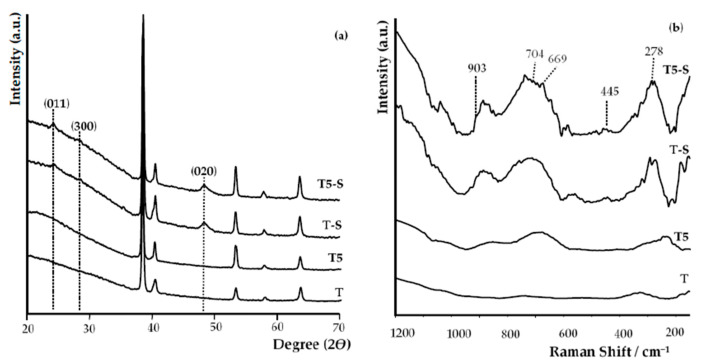
X-ray diffraction spectra of T, T5 samples without alkali-sodium treatment and T-S, T5-S samples with alkali-sodium treatment (**a**), where marked peaks are assigned to sodium titanate; Raman spectra of T, T5 samples without alkali-sodium treatment and T-S, T5-S samples with alkali-sodium treatment (**b**).

The strong signals at 24° and 48°, and weak signal at 28° (Figure 3a), which we found in XRD patterns of T-S and T5-S samples, were attributed to (011), (020), and (300) lines of sodium titanate crystals, respectively. Positions of these peaks are in good agreement with the literature data for sodium trititanate gel (Na_2_Ti_3_O_7_) [25,54,55,56,57,58,59,60]. Moreover, the above-mentioned EDX data of oxygen and titanium mass percentage for T-S (Figure 2c) are in good agreement with the theoretical elemental mass percentage for sodium trititanate (O experimental 37%, theoretical 39.9%; Ti experimental 47%, theoretical 50.7%). The formation of sodium titanates was also confirmed by the analysis of Raman spectra (Figure 3b, Table 1)).

Raman spectra analysis of alkali-sodium-treated samples confirmed the sodium titanate formation, as evidenced by the appearance of five bands at c.a. 278 cm^−1^, 445 cm^−1^, 668 cm^−1^, 704 cm^−1^, and 903 cm^−1^ (Figure 3b) [25,32,43,61,62]. The bands which appeared at ∼903 cm^−1^ and ~278 cm^−1^ are characteristic for sodium trititanates (Na_2_Ti_3_O_7_) and they are not visible in spectra of hydrogen titanates [25,61,62,63,64,65]. The XRD patterns and Raman spectra were also used in order to deduce the amorphous structure of produced T5 coatings (Figure 3a).

The results of wettability studies of studied coatings are presented in Table 2. The water contact angle for the unmodified T sample was approximately 81.3 ± 0.2°, and that for T5 was 94.4 ± 0.4°. These samples indicated a less and more clear hydrophobic character, respectively. After alkali-sodium treatment, a drop of both water and diiodomethane spread rapidly and wetted the treated samples of T5-S and T-S. The water and diiodomethane contact angle decreased to nearly 0°; thus, the surface of these samples can be considered completely wetted, which means that these surfaces are amphiphilic.

### 3.2. Apatite-Forming Ability

**Figure 4:**
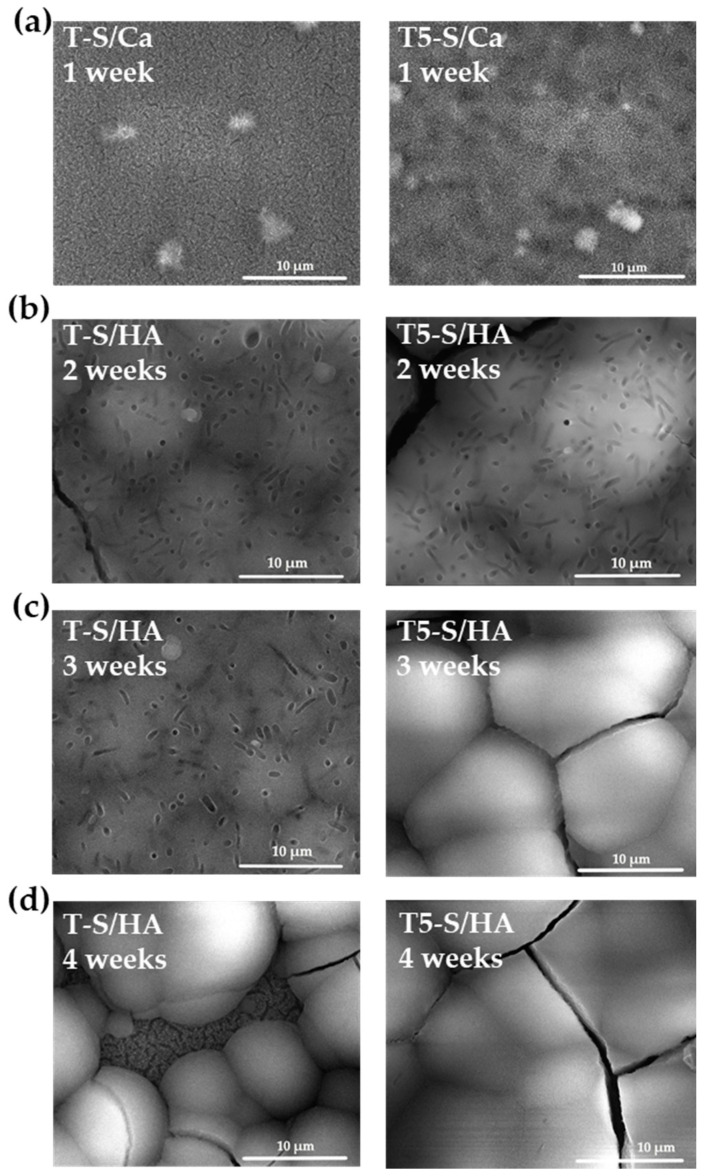
Scanning electron microscopy (SEM) images of substrates of T-S and T5-S with CaTiO_3_ (Ca) or hydroxyapatite (HA) layers after immersion in SBF for 1 (**a**), 2 (**b**), 3 (**c**), and 4 (**d**) weeks.

**Figure 5:**
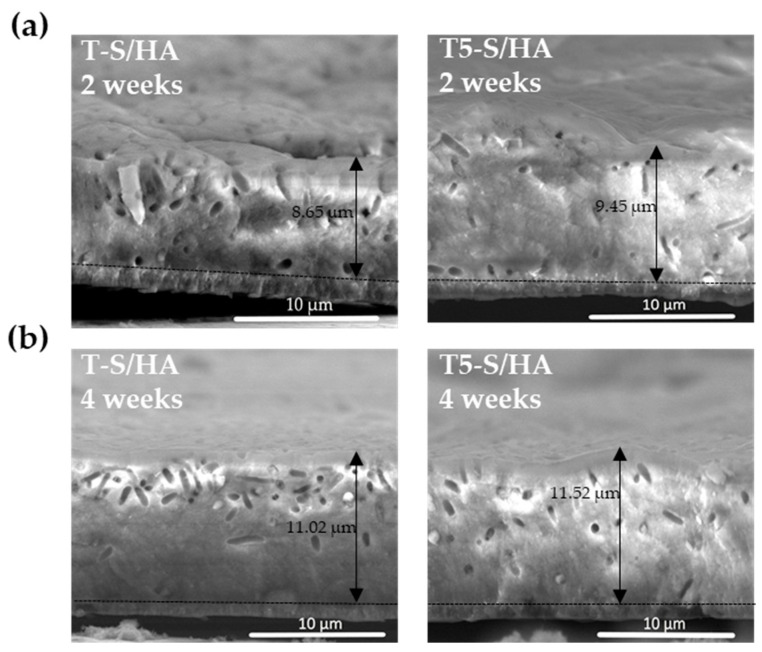
SEM cross-section images of T-S and T5-S with hydroxyapatite (HA) layers after immersion in SBF for 2 (**a**) and 4 (**b**) weeks. A horizontal black line separates the hydroxyapatite (HA) layer from T-S and T5-S coatings.

A basic assay indicating the potential bioactivity of a material is its incubation in SBF solution containing calcium and phosphorus ions and apatite precipitation on the surfaces of tested materials. Figure 4 shows the SEM top view images of T-S and T5-S after their immersion in SBF solution for 7, 14, 21, and 28 days. Figure 5 shows SEM cross-section images of T-S and T5-S with hydroxyapatite (HA) layers after immersion in SBF for 2 and 4 weeks. The aim of these studies was to monitor the development of apatite formation on their surfaces. The materials marked as T-S/Ca and T5-S/Ca represent samples containing calcium titanates (CaTiO_3_) on the surface, which was formed after 7 days of sample immersion in SBF. On the other hand, the materials marked as T-S/HA and T5-S/HA represent samples containing hydroxyapatite on the surface, which was created after longer sample immersion in SBF.

**Figure 6:**
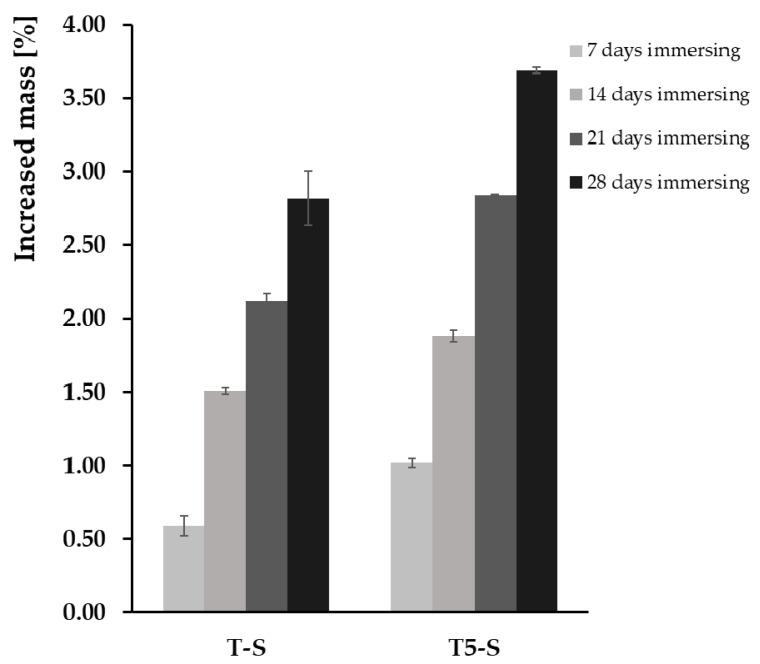
Weight gain of T-S and T5-S with CaTiO_3_ (Ca) or hydroxyapatite (HA) layers after immersion in SBF for 7, 14, 21, and 28 days.

The percentage weight gain that was observed after removing and drying the samples from the SBF solution is presented in Figure 6. On the surface of T and T5 control samples, formation of apatite was not observed. On the other hand, the SEM images of T-S samples, immersed in SBF solution for 1 week, show mostly nanofibers and poorly observed small agglomerated globules mainly of Ca and P on the sample surfaces (Figure 4a). Analysis of these data revealed an increase in the sample weight after 14 days of sample immersion in SBF solution (Figure 4b,c). The T-S surface was partially covered with a thick hydroxyapatite layer after 28 days of immersion (Figure 4d). In the case of T5-S, the formation and growth of dispersed particles consisting of calcium and phosphorus were observed after 7 days of layer immersion in the SBF solution, which was confirmed by the EDS analysis (Figure 4a). The mass increase for the T5-S sample was ca. 1.7 times higher than that for T-S. After 14 days of immersion in SBF, hydroxyapatite was formed on the surface of T5-S specimens, with a ca. 1.31-fold increase in the sample mass observed compared to that of T-S (Figure 5 and Figure 6). Considering the results obtained, it should be noted that the formation of sodium titanate layers on T and T5 substrates promoted apatite formation, while earlier anodization of the Ti6Al4V substrate surface (T5-S) was more favorable for the growth of apatite.

The desired effect is to obtain, on the surface of apatite, a structure as close as possible to natural hydroxyapatite (HA) with a Ca/P molar ratio of 1.67. The Ca/P molar ratio of titanate layers was investigated through EDS analysis, and is the results are shown in Table 3. Phosphorus and calcium were the main elements detected in the specimens, allowing the Ca/P molar ratio to be estimated. The presence of sodium and magnesium ions was also observed in small amounts. Sodium probably remained after alkali-sodium surface modification. After 1 week in the alkali samples, the detected Ca/P molar ratio was 7.26 and 8.98 for T-S/Ca and T5-S/Ca, respectively. After the second and third weeks, this ratio decreased rapidly for T5-S/HA to 1.76 and 1.84, respectively. For T-S/HA, after the second and third week, the Ca/P ratio also decreased, although it was higher than that for T5-S/HA, with values of 1.94 and 2.00, respectively. According to the literature, it can be assumed that, since there are no stable calcium phosphate compounds known to exhibit a molar ratio Ca/P higher than 2, these elements occurred as ions in the titanate surface. After 4 weeks of sample immersion in SBF solution, the Ca/P molar ratios of 1.84 for T5-S/HA and 1.82 for T-S/HA were detected.

**Figure 7:**
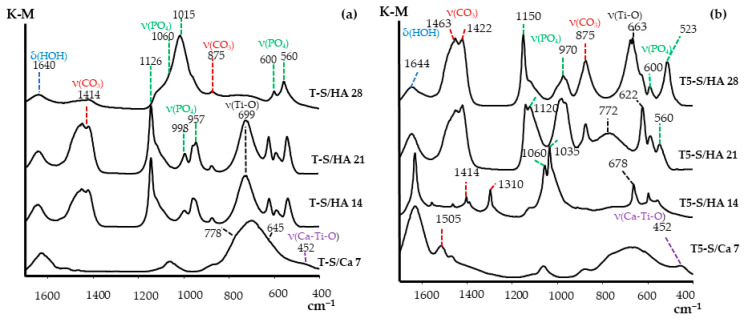
Diffuse reflectance infrared Fourier transform spectroscopy (DRIFT) spectra of studied T-S/Ca and T-S/HA (**a**) and T5-S/Ca and T5-S/HA (**b**) after immersion in SBF for 7, 14, 21, and 28 days.

The DRIFT spectra of all studied samples, after immersion in SBF solution for 1–4 weeks, are shown in Figure 7. The intense bands attributed to phosphate groups of HA were found at 1150–1015 (ν_3_(PO_4_)), 970 (ν_1_(PO_4_)), 600 (ν_4_(PO_4_)), 560 (ν_4_(PO_4_)), and 523 (ν_4_(PO_4_)) cm^−1^ for T5-S/HA and T-S/HA samples after their immersion for 14, 21, and 28 days [3,30,66,67,68,69,70,71,72,73,74,75,76]. The band detected at 452 cm^–1^ in the spectra of T5-S/Ca 7 and T-S/Ca 7 was attributed to the Ca–Ti–O group modes [77,78]. A weak band at ca. 1060 cm^−1^ in the studied spectra indicated the presence of P–O functional groups. The strong bands, which were registered in the spectra of T5-S/HA 21 and T5-S/HA 28 samples between 1400 and 1505 cm^−1^ (stretching mode of ν_1_ (CO_3_^2−^) group in B-type carbonate HA and bending mode of (ν_3_ or ν_4_) CO_3_^2−^ group in carbonate HA at 875 cm^−1^) were assigned to surface carbonate ions, which are commonly found in both synthetic HA and natural bone [3,30,33,67,69,70,71,72,74,75,76]. However, the characteristic bands at 875 cm^−1^ indicated the presence of HPO_4_^2−^ in the crystal lattice [67,70,76]. The band at 1310 cm^−1^ (ν_3_), appearing in the spectrum of T5-S/HA 14 (i.e., after 14 days of immersion in SBF), was assigned to surface carbonate ions [71,72,73]. Since the samples after removal from the SBF solution were not dried at higher than room temperature, we can assume that the carbonate bands were in a non-quantity position. The band at 1640–1644 cm^−1^ can be assigned to ν_3_ modes of the carbonate ion; however, on the other hand, it may be a bending mode, attributed to H_2_O in the SBF-treated titanate layer [70,74,76]. Figure S1 (Supplementary Materials) shows the stretching bands between 3000 and 3600 cm^–1^, which can be related to intense and broad bands of O–H stretching in the HA structure and to adsorbed water molecules. These –OH bands were present in all the spectra collected from the sample surface layer. The wide bands attributed to the Ti–O vibration were observed between 640 and 780 cm^−1^ [3,30,33,66,67,68,70,71,72,76].

**Figure 8:**
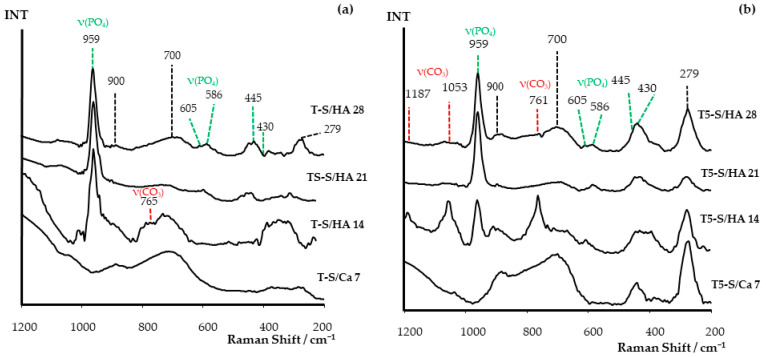
Raman spectra of studied (**a**) T-S/Ca and T-S/HA and (**b**) T5-S/Ca and T5-S/HA after immersion in SBF for 7, 14, 21, and 28 days. The undescribed peaks came from titanate.

The apatite-forming ability was also confirmed by the Raman spectra (Figure 8). A strong band, attributed to symmetric stretching P–O vibrations (ν_1_; active in Raman spectrum), was found at 959 cm^−1^ in the spectra of T5-S/HA and T-S/HA samples after 14, 21, and 28 days [3,25,79,80]. On the other hand, the weak bands at ~586 cm^−1^ and 605 cm^−1^, detected in the spectra of all samples, were assigned to ν_4_ P–O bending modes [3,79,80]. The bending modes (ν_2_) bands of HA PO_4_ groups were registered at 430 cm^−1^ and 445 cm^−1^ in the spectra of T-S/HA 14–28 and T5-S/HA 14–28 samples. However, in the spectra of T5-S/Ca 7 samples, bands attributed to O–Ti–O bending vibrations could also be found in abovementioned spectral range [3,81,82]. The bands, appearing at 1187 cm^−1^, 1053 cm^−1^, and 761 cm^−1^, confirmed the presence of CO_3_^2−^ ions in the T5-S/HA 14 sample [3,83]. The detection of the band at 279 cm^−1^ in the Raman spectra of studied samples confirmed bond formation among the sodium, calcium, and O–Ti–O group. The Raman spectrum of CaTiO_3_ included a number of sharp bands imposed on a broad feature between 240 cm^−1^ and 370 cm^−1^, and they were tentatively assigned to the modes associated with rotations of the oxygen cage [81,84,85,86]. This was clearly noticeable for the T-S/Ca 7 sample.

**Figure 9:**
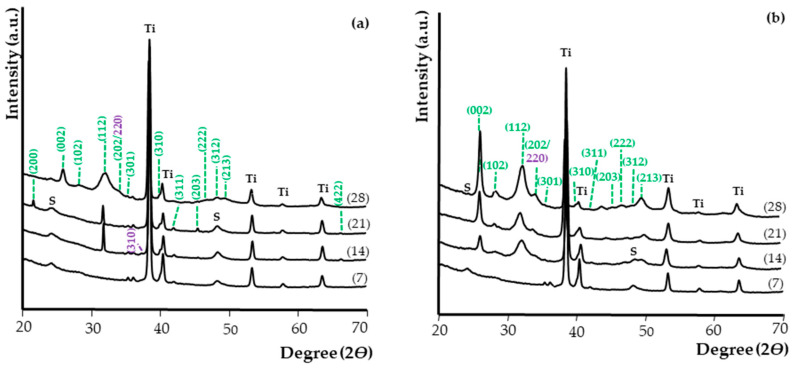
X-ray diffraction patterns of T-S/Ca (7) and T-S/HA (14–28) (**a**) and T5-S/Ca (7) and T5-S/HA (14–28) (**b**) samples after immersion in SBF solution for 7, 14, 21, and 28 days. (*hkl*) for HA are marked in green, while (*hkl*) for CaTiO_3_ are marked in violet. Ti was assigned to the Ti6Al4V substrate; S was assigned to the sodium titanate.

The apatite presence on the surface of samples immersed in SBF was confirmed by X-ray diffraction (XRD). Figure 9 shows the XRD spectra of T-S/Ca and T-S/HA (a) and T5-S/Ca and T5-S/HA (b) samples after immersion in SBF for 1–4 weeks. The XRD results were consistent with the SEM images, as well as EDS, Raman, and DRIFT analyses. It was noted that, after immersing the samples in SBF solution, on the surface of T-S and T5-S layers, hydroxyapatite was formed after 14 days. Simultaneously, with the increasing immersion time of layers in SBF, the amount of apatite grew. The presence of peaks attributed to calcium titanate confirmed that the alkali-sodium titanate layers released alkali ions into the SBF solution via exchange with the H_3_O^+^ ions. In effect, Ti–OH groups were formed. These hydrated titania groups induced apatite nucleation, which proceeded to calcium titanate (CaTiO_3_) formation and combination with phosphate ions. The apatite nuclei were spontaneously grown on layers by consuming the Ca^2+^ and PO_4_^3−^ ions from the surrounding fluid. As a result of this process, apatite was formed, which contained ions such as carbonate, sodium, and magnesium.

### 3.3. Cell Viability Measured with the MTT Assay

**Figure 10:**
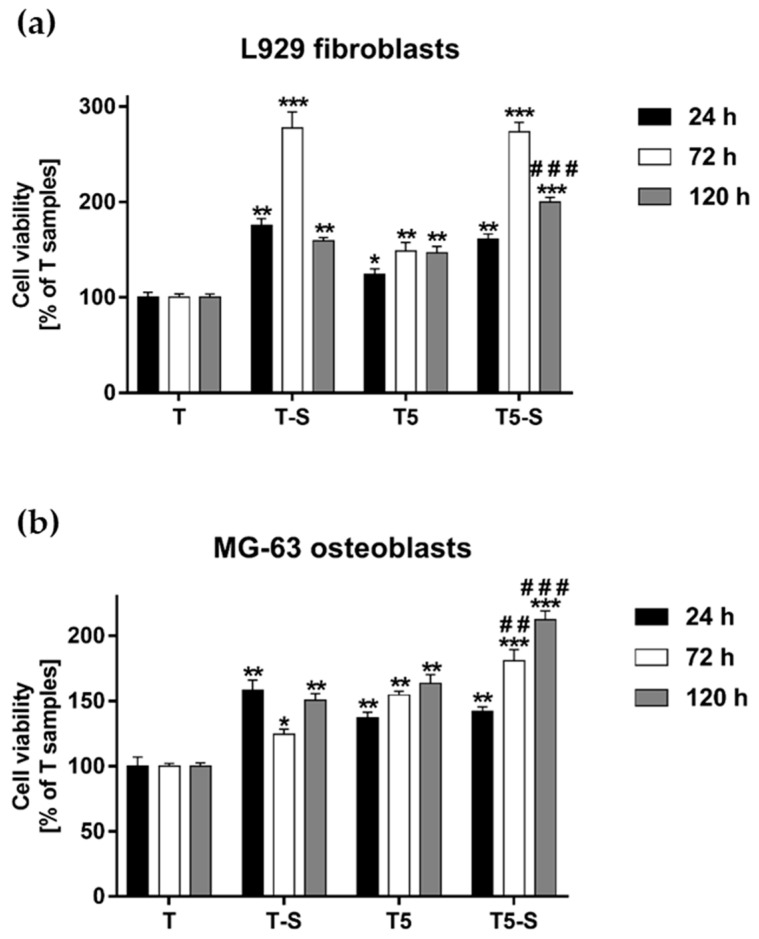
Cell viability of L929 fibroblasts (**a**) and MG-63 osteoblasts (**b**) cultured on the T-S, T5- S, and T5 samples for 24, 72, and 120 h. The results were compared with the reference Ti6Al4V samples (T). The cell viability is presented as a percentage of the control cells growing on the T specimens (served as 100%). Data are presented as the mean ± standard error of the mean (SEM). Asterisks denotes differences when the viability of cells growing on the tested scaffolds was higher compared with T samples (*** *p* < 0.001, ** *p* < 0.01, * *p* < 0.05). The pound signs indicate differences between the cells cultured on the T5-S scaffolds and T-S samples (### *p* < 0.001, ## *p* < 0.01).

The viability of L929 fibroblasts and MG-63 osteoblast-like cells growing on the scaffolds was assessed using the MTT assay. The results demonstrated that all tested samples induced a higher cell viability than the reference Ti6Al4V alloy foils (T), as observed after 24, 72, and 120 h of incubation time (Figure 10). Moreover, it was found that T-S and T5-S scaffolds increased the viability of L929 fibroblasts compared with not only the reference alloy, but also T5 samples. This effect was noticed especially after 72 h of incubation time (Figure 10a). Among all the tested samples, T5-S scaffolds induced the highest viability of MG-63 cells after 72 and 120 h of culture (Figure 10b). Importantly, T5-S samples also increased the viability of L929 cells in comparison with T-S specimens after 120 h of culture (*p* < 0.001).

**Figure 11:**
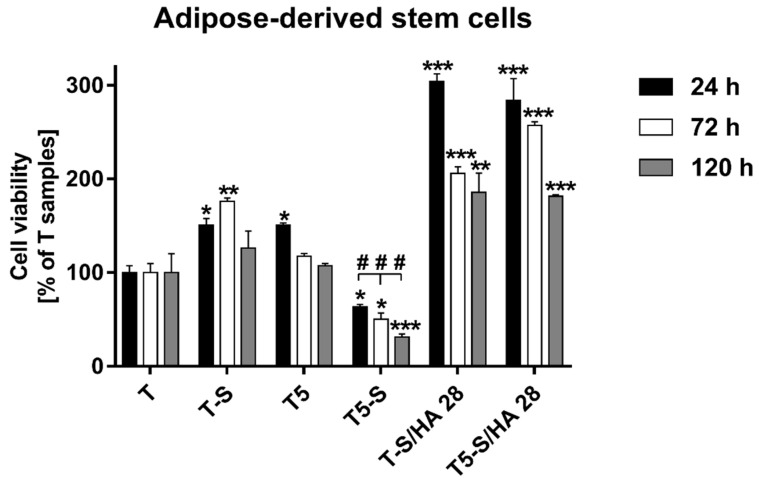
Viability of adipose-derived mesenchymal stem cells (ADSCs) cultured on the tested samples for 24, 72, and 120 h. The results are expressed as the mean ± SEM and presented as a percentage of the cells cultivated on the T samples (served as 100%). Asterisks indicate differences when the viability of cells growing on the tested scaffolds was higher in comparison with T samples (*** *p* < 0.001, ** *p* < 0.01, * *p* < 0.05). Pound signs denote differences between the cells cultured on the T5-S scaffolds and T-S samples (### *p* < 0.001).

The viability of ADSCs cultured on the tested specimens varied significantly between fibroblasts and osteoblasts, as well as between both alkali-sodium-modified scaffolds (Figure 11). The T-S sample promoted an approximately 50% higher level of ADSC proliferation in comparison with Ti6Al4V reference alloy foils (T), after 24, 72, and 120 h of incubation time. Surprisingly, it was found that T5-S scaffolds completely inhibited the proliferation of ADSCs. The cells adhered weaker to the surface, and cell viability was considerably decreased in relation to T reference alloy foils. On the contrary, the hydroxyapatite layer greatly improved the surface properties. ADSCs exhibited strong adhesion on T-S/HA 28 and T5-S/HA 28 specimens, and their number during the first 24 h of culture increased threefold in comparison to the control. After 72 and 120 h, the proliferation rate decreased; however, the cell viability compared to the reference T specimen still increased approximately twofold.

### 3.4. Cell Morphology Observed by Scanning Electron Microscopy

**Figure 12:**
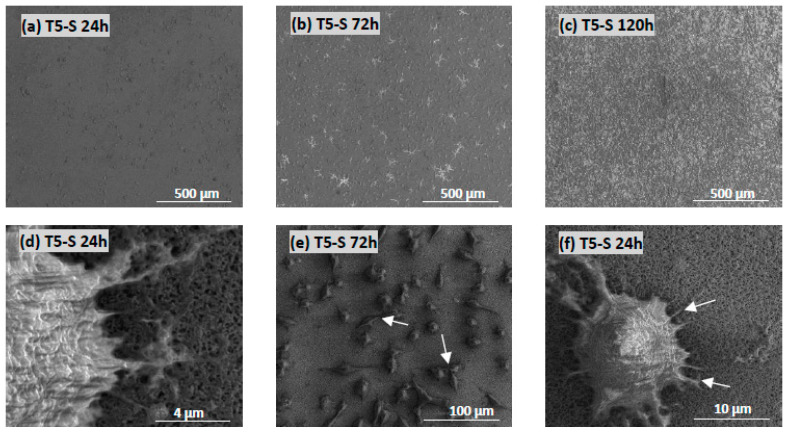
Images obtained by SEM showing L929 fibroblasts that were cultured on the T5-S scaffolds for 24 (**a**), 72 (**b**), and 120 h (**c**). Arrows present the filopodia spreading between fibroblasts (**e**) and the filopodia that attached the cell to the surface of scaffolds (**d**,**f**).

**Figure 13:**
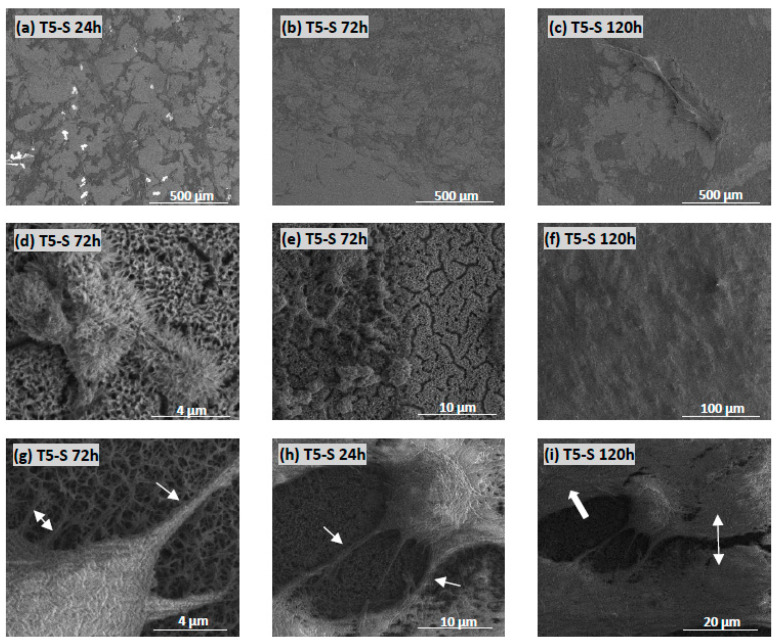
Micrographs obtained by SEM showing MG-63 osteoblast-like cells cultured on the T5-S specimens for 24 (**a**), 72 (**b**), and 120 h (**c**). Figures (**d**–**f**) indicate that the MG-63 cells are morphologically similar to the structure of T5-S samples. Arrows in the micrographs present filopodia which attached the cell to the substrate (**g**) or the filopodia created between the cells (**h**). Figure (**i**) indicates the extracellular matrix produced by osteoblast-like cells.

**Figure 14:**
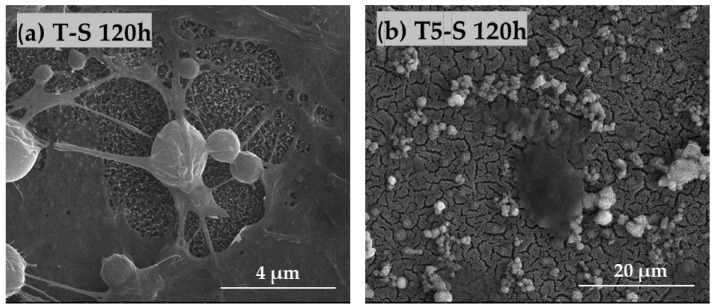
Comparative SEM images showing human ADSCs cultured on the surface of T-S (**a**) and T5-S (**b**) specimens for 120 h. The figures confirm evident differences in cell morphology for both samples: adherent cells with filopodia attached to the surface of T-S (**a**) and rounded cells without attachments on T5-S (**b**).

The morphology and proliferation level of L929 fibroblasts (Figure 12) and MG-63 osteoblast-like cells (Figure 13) growing on the surface of T5-S specimens was demonstrated in the micrographs produced by scanning electron microscopy (SEM). These images confirmed the results from the MTT assay and showed the increase in cell proliferation level over time, as observed for both tested cell lines (compare micrographs (a–c) in Figure 12a–c and Figure 13). The fibroblasts and osteoblasts had an elongated shape, and they effectively attached to the scaffolds by forming numerous filopodia (Figure 12d, and arrows in Figure 12f and Figure 13g), which were also generated between the cells (arrows in Figure 12e and Figure 13h). As can be seen in Figure 12c and Figure 13c, most of the T5-S sample surface was covered with the cells. Moreover, we also noticed that it was difficult to observe MG-63 osteoblast-like cells cultured on the specimens since the surface of nanocoatings morphologically resembled the cells (Figure 13d–f). MG-63 cells also produced an extracellular matrix, as can be observed in Figure 13i. On the contrary, ADSCs grown on the T-S surface adhered properly and produced an extracellular matrix (Figure 14a), whereas the surface of T5-S did not support ADSC growth (Figure 14b). Comparative SEM images also revealed some differences in the morphology of ADSCs cultured on the T5-S surface specimens for 120 h (Figure 14b). The cells growing on T-S scaffold created filopodia that attached the cells to the substrate, whereas ADSCs cultured on T5-S samples had a rounded shape without attachments.

## 4. Discussion

A crucial stage in the design of modern implants is to modify their surfaces, ensuring the proper interaction of the implants with the tissue environment. In this study, we focused on the modification of the Ti6Al4V and Ti6Al4V/nanoporous TiO_2_ substrate surface by the production of titanate layers using the alkali-sodium treatment method, which is known as an effective and simple procedure.

The starting point for the discussion of received results was the evaluation of the bioactivity of Ti6Al4V (T) substrates, whose surface was modified by an alkali-sodium treatment at 65 °C for 48 h (T-S). The MTT assays proved that adsorption of L929 fibroblast cells and MG-63 osteoblast-like cells (after 24 h) and their proliferation (after 72 and 120 h) explicitly increased for samples after their surface modification (Figure 10). The formation of the Na_2_Ti_3_O_7_ nanofibrous layer on the T and T5 surfaces led to an increase in surface area, thereby promoting the formation of apatite in body fluids (SBF). Analysis of SEM, EDS, XRD, and Raman data (Figure 1 and Figure 3) confirmed the sodium trititanate layer formation both on the T substrate (T-S) and on the T5 coating (T5-S) after hydrothermal treatment in a 7 M NaOH solution. These results are in good agreement with the observation of Zhang et al. [58]. Moreover, Fatehi et al. [49] showed that, with increasing concentration of NaOH solution, time, and temperature of alkaline treatment, the thickness of titanate gel layer increased. However, according to abovementioned reports, the use of an NaOH solution concentration higher than 10 M could lead to the delamination of the thick sodium titanate layer from the titanium substrate. The use of the 7 M NaOH solution (in our experiments) did not cause the delamination of the titanate layer from the surface of T-S and T5-S substrates, which was confirmed by the analysis of SEM images. Rahimipour et al. [87] carried out alkaline etching of the NiTi alloy substrate using 10 M NaOH solution. The samples were etched at *T* = 60, 120, or 180 °C for 48 h. According to this report, with the increase in temperature, sodium and oxygen concentration increased, which indicated the growth of formed surface layers and allowed determining the surface morphology. The influence of temperature on the increase of the titanate layer was not studied in our experiments; however, on the basis of an analysis of the EDS data, we can observe that the appearance of Na and O on the surface of T-S and T5-S samples led to a significant increase in the thickness of formed titanate layers. Jiandi et al. drew attention on the clear increase in sodium titanate gel layer thickness with the immersion time of Ti6Al4V samples, which were immersed in 8 M NaOH solution at 60 °C for 12, 24, 36, 48, and 60 h [88]. A similar effect was noticed in this study for Na_2_Ti_3_O_7_ nanofibrous layers produced on the surface both T-S and T5-S samples. It should be noted that earlier anodization of Ti6Al4V substrates induced the formation of a thicker titanate layer in comparison to the reference sample T. The nanofibrous architecture of produced layers can significantly increase the substrate surface area and, thus, provide more nucleation sites for hydroxyapatite anchored in the substrate [88].

One of the ways to evaluate the bioactivity of a material is to study the spontaneous formation of apatite on its surface, while it is immersed in a similar physiological medium [89], which is due to fact that the bone partially consists of non-stoichiometric calcium phosphate [30]. Therefore, very important for us was to determine the relationship between the different surface characteristics of materials and the bioactivity of materials (apatite-forming ability). As stated above, in vitro studies in SBF solution showed faster apatite formation on the surface of T5-S than T-S. This may be the result of differences in the morphology of these samples, as well as the increased surface area. The surface of T5 after anodization was composed of titanium dioxide, and, in this form, it was treated with alkali-sodium (T5-S). On such a large surface, a number of Ti–OH groups were formed and calcium ions were incorporated into the hydrated Ti–OH layer to form calcium titanate and to stimulate the growth of apatite nucleation [28,38]. An important part of T-S and T5-S investigations was the determination of their wettability via liquid drop analysis on the surfaces. Both samples revealed amphiphilic behavior, attracting both polar and dispersive media (Table 2). Wang et al. [25] reported that a drop of water spread quickly and wetted alkaline-treated titanium samples, with a water contact angle close to 0° (superhydrophilic character). Kazek-Kęsik et al. [90] also showed that the use of the alkali surface treatment process resulted in a significant reduction in water contact angle in contact with the Ti-15Mo, Ti-13Nb-13Zr, and Ti-6Al-7Nb alloys. It is assumed that the hydrophilic surface of dental implants enhances the adhesion and orientation of selected proteins. Our research broadens this information, as we also decided to investigate the nature of T-S and T5-S sample surfaces in reaction with a drop of hydrophobic liquid. In this way, we additionally proved the amphiphilic character of the samples after alkali treatment.

Alkali-sodium treatment of Ti6Al4V (T) and Ti6Al4V/TiO_2_ (T5) improved, in general, their biocompatible properties. The T-S sample promoted the cell growth and proliferation of all tested cell lines (fibroblasts, osteoblasts, and mesenchymal stem cells), whereas T5-S was beneficial only for fibroblasts and osteoblasts. The proliferation level of MG-63 osteoblast-like cells growing on the T5-S specimen was significantly higher than that on the T control sample. Conversely, the osteoblast-promoting scaffold entirely reduced the proliferation of ADSCs. It can be concluded that surface anodization followed by alkali-sodium treatment favors the growth of osteogenic lineage cells. This conclusion was additionally supported with micrographic analyses. Osteoblasts growing on the surface of alkali-sodium-modified specimens showed a significant degree of integration with the scaffold, which is of great importance for the development of implants. Considering that new implant surfaces are developed to improve biological cell responses, guiding the differentiation of mesenchymal stem cells toward osteoblasts and enhancing osseointegration, it is commonly reported in the most recent literature that cell growth is facilitated on rough substrates with diversified topography [3,91,92,93,94]. Additionally, effective osseointegration is significantly stimulated with increasing surface roughness of implants, inter alia titanium substrates (reviewed in [95]). Increased roughness induces beneficial morphological changes of growing cells and production of extracellular matrix components, together with positive interplay with the surrounding tissues. The limitations in preparing such surfaces include the possibility of reduced strength of the roughened material. Therefore, in the case of modifications used for long-term implants, it would be highly desirable to prepare surfaces with a roughness on the micrometer scale [94].

The ability to form apatite on the surface of T-S and T5-S also considerably improved the surface properties in terms of ADSC adhesion and proliferation. As apatite or similar mineralized structures create an osteogenic microenvironment that is extremely valuable for osseointegration, it can be assumed that ADSCs undergo differentiation, which was reflected in the lowered proliferation rate after 72 and 120 h of culture on T-S/HA and T5-S/HA. This remains in agreement with other in vitro studies on modified HA scaffolds, which have proven the capability of ADSCs to adhere and colonize the material and to undergo differentiation to an osteogenic lineage with subsequent scaffold mineralization, even without the addition of osteo-inductive factors to the cell medium [96,97].

## 5. Conclusions

The research carried out exhibited that alkaline treatment of Ti6Al4V (T) substrates and Ti6Al4V covered by a nanoporous TiO_2_ coating (T5) led to the formation of Na_2_Ti_3_O_7_ nanofibrous layers (T-S and T5-S), whose thickness depended on the immersion time. It should be noted the clear influence of the anodization process of the Ti6Al4V substrates on the thicker sodium titanate layer formation (T5-S) in comparison to the non-anodized substrate (T-S). The anodization of the T surface was also important for increasing the ability to form apatite. Analysis of MTT assays revealed that surface anodization followed by alkali-sodium treatment could be important for implant design and production. The development of scaffolds with surface properties mimicking the bone structure and capable of inducing the right commitment of seeded cells is still a challenge. We believe that our results represent an important advancement in this context.

## 6. Patents

The patent application was registered at the Patent Office of the Republic of Poland; P.435368; [WIPO ST 10/PL435368]; Ehlert, M.; Piszczek, P.; Radtke, A.; A method of producing a nanocomposite coating on the surface of a Ti6Al4V titanium alloy and the coating produced by this method.

## Figures and Tables

**Table 1:** Raman frequencies and assignments of functional groups in sodium titanate samples.

Functional Groups	Frequencies (Experimental) (cm^−^^1^)	Frequencies (Reference) (cm^−^^1^)	Reference
ν (Na–O–Ti)	278	280	[61,62,63]
δ (Ti–O)	445	440, 448	[43,63]
ν (Na–O–Ti)	669	660	[32,63]
δ (Ti–O)	704	690, 700	[43,63]
ν (Ti–O–Na)	903	905, 920	[43,61,63]

**Table 2:** The values of contact angles for water and diiodomethane of the T, T5, T-S, and T5-S samples.

Biomaterial Sample	Average Contact Angle (°) ± Standard Deviation	
	Measuring Liquid	
	Water	Diiodomethane
T	81.3 ± 0.2	49.2 ± 0.9
T5	94.4 ± 0.4	22.4 ± 1.0
T-S	˂10	˂10
T5-S	˂10	˂10

**Table 3:** Ca/P ratios obtained from EDS measurements for the T, T5, T-S, and T5-S samples after immersion in SBF for 1–4 weeks.

Biomaterial Sample	Ca/P (Molar Ratio) after 7 Days	Ca/P (Molar Ratio) after 14 Days	Ca/P (Molar Ratio) after 21 Days	Ca/P (Molar Ratio) after 28 Days
T	No growth	No growth	No growth	No growth
T5	No growth	No growth	No growth	No growth
T-S	7.26	1.94	2.00	1.82
T5-S	8.98	1.76	1.84	1.84

## Data Availability

Data sharing is not applicable in this article.

## Supplementary Materials

The following are available online at https://www.mdpi.com/1996-1944/14/4/806/s1. Figure S1: DRIFT spectra (1600–4000 cm^−1^) of studied T-S/Ca and T-S/HA (**a**) and T5-S/Ca and T5-S/HA (**b**) after immersion in SBF for 7, 14, 21, and 28 days.
