# Supplementary material for: Assessment of Titanate Nanolayers in Terms of Their Physicochemical and Biological Properties
Source: Materials (Basel). 2021 Feb 8;14(4):806. doi: 10.3390/ma14040806 (PMC7915217; doi:10.3390/ma14040806)
Supplement: Supplementary file 1 [file materials-14-00806-s001.pdf]

materials

Supplementary Materials

Assessment of Titanate Nanolayers in Terms of Their Physicochemical and Biological Properties

Michalina Ehlert [1,2], Aleksandra Radtke [1,2,*], Katarzyna Roszek [3], Tomasz Jędrzejewski [3] and Piotr Piszczek [1,2,*]

[1] Faculty of Chemistry, Nicolaus Copernicus University in Toruń, Gagarina 7, 87-100 Toruń, Poland; m.ehlert@doktorant.umk.pl
[2] Nano-implant Ltd., Gagarina 5/102, 87-100 Toruń, Poland
[3] Faculty of Biological and Veterinary Sciences, Nicolaus Copernicus University in Toruń, Lwowska 1, 87-100 Toruń, Poland; kroszek@umk.pl (K.R.); tomaszj@umk.pl (T.J.)
* Correspondence: aradtke@umk.pl (A.R.); piszczek@umk.pl (P.P.); Tel.: +48-60-032-12-94 (A.R.); +48-60-788-33-57 (P.P.)

Citation: Ehlert, M.; Radtke, A.; Roszek, K.; Jędrzejewski, T.; Piszczek, P. Assessment of Titanate Nanolayers in Terms of Their Physicochemical and Biological Properties. *Materials* **2021**, *14*, 806. https://doi.org/10.3390/ma14040806

Academic Editor: Carla Renata Arciola

Received: 5 January 2021
Accepted: 1 February 2021
Published: 8 February 2021

Publisher's Note: MDPI stays neutral with regard to jurisdictional claims in published maps and institutional affiliations.

Copyright: © 2021 by the authors. Licensee MDPI, Basel, Switzerland. This article is an open access article distributed under the terms and conditions of the Creative Commons Attribution (CC BY) license (http://creativecommons.org/licenses/by/4.0/).

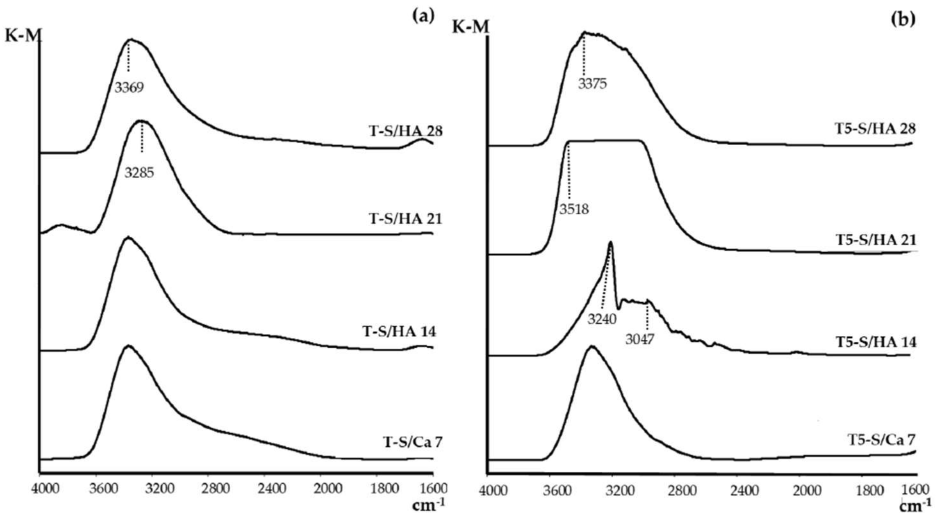

Figure S1. DRIFT spectra (1600–4000 cm^{-1}) of studied T-S/Ca and T-S/HA (**a**), T5-S/Ca and T5-S/HA (**b**) after immersing in SBF for 7, 14, 21 and 28 days.
